# The immune response of stem cells in subretinal transplantation

**DOI:** 10.1186/s13287-015-0167-1

**Published:** 2015-09-14

**Authors:** Bikun Xian, Bing Huang

**Affiliations:** State Key Laboratory of Ophthalmology, Zhongshan Ophthalmic Center, Sun Yat-sen University, Guangzhou, 510060 Guangdong Province China

## Abstract

Stem cell transplantation is a potential curative treatment for degenerative diseases of the retina. Among cell injection sites, the subretinal space (SRS) is particularly advantageous as it is maintained as an immune privileged site by the retinal pigment epithelium (RPE) layer. Thus, the success of subretinal transplantation depends on maintenance of RPE integrity. Moreover, both embryonic stem cells (ESCs) and mesenchymal stem cells (MSCs) have negligible immunogenicity and in fact are immunosuppressive. Indeed, many studies have demonstrated that immunosuppressive drugs are not necessary for subretinal transplantation of stem cells if the blood-retinal barrier is not breached during surgery. The immunogenicity of induced pluripotent stem cells (iPSCs) appears more complex, and requires careful study before clinical application. Despite low rates of graft rejection in animal models, survival rates for ESCs, MSCs, and iPSCs in retina are generally poor, possibly due to resident microglia activated by cell transplantation. To improve graft survival in SRS transplantation, damage to the blood-retinal barrier must be minimized using appropriate surgical techniques. In addition, agents that inhibit microglial activation may be required. Finally, immunosuppressants may be required, at least temporarily, until the blood-retinal barrier heals. We review surgical methods and drug regimens to enhance the likelihood of graft survival after SRS transplantation.

## Introduction

Retinal degenerative diseases such as age-related macular degeneration and retinitis pigmentosa are characterized by irreversible loss of retinal pigment epithelium (RPE) cells, photoreceptors, choriocapillaries, and other retinal cells [1]. These degenerative diseases afflict millions of people worldwide but there are currently no broadly effective treatments for them. Stem cell-based therapy is a potential approach for the treatment of retinal degenerative diseases [2–7], and many animal studies [8–15] and some clinical trials [16–18] have indeed shown encouraging results.

Stem cells have the capacity for both self-renewal and differentiation into multiple cell lineages (pluripotency) [19]. They also have low immunogenicity [20, 21], reducing the chance of rejection. Moreover, the subretinal space (SRS) is an immunoprivileged site within the eye [22] and thus a logical target for cell transplantation. In fact, grafts transplanted into the SRS have shown better migration and integration than cells transplanted into the vitreous cavity [23, 24]. Transplantation of low-immunogenicity cells into this immunoprivileged site appears particularly promising, although most studies using stem cells for SRS transplantation have observed poor survival. Successful transplantation thus requires more detailed knowledge of how the host immune system responds to SRS stem cell transplantation. In this review, we discuss the immune characteristics of the SRS and of various stem cells, and their interaction after transplantation. We then summarize methods that can suppress the immune response of the host and improve graft survival.

## Immune privilege of the subretinal space

The SRS is the area between the RPE layer and the outer limiting membrane of the retina. The RPE is critical for the immunoprivileged status of the SRS. Sugita *et al.* [25] found that RPE cells *in vitro* can suppress T-cell activation by direct cell-to-cell contact, and Zamiri and colleagues [26] reported that just the supernatant of the RPE eyecup suppressed T-cell activation and production of interferon. Wenkel and Streilein [27] reported that the SRS suppressed cell-associated and soluble antigen-specific delayed-type hypersensitivity. Moreover, they found that the immune privilege of the SRS was dependent on the presence of an intact and healthy RPE cell monolayer. The mechanisms conferring immune privilege include (1) suppression of T-cell activation by release of cytokines from the RPE, such as transforming growth factor-β [28], thrombospondin-1 [29], prostaglandin E2 [25], cytotoxic T lymphocyte associated antigen-2α [30], and retinoic acid [31]; (2) production of other immunosuppressive factors by RPE cells that suppress innate immune activity, including pigment epithelium-derived factor and somatostatin [32]; (3) surface expression of program death-1 (PD-L1) [33] and Fas ligand [34] by RPE cells; (4) conversion of CD8+ and CD4+ T cells into regulatory T cells [30]; and (5) the intact physical barrier of the RPE layer [27].

## The immunogenicity of stem cells

Stem cells hold great promise for regenerative medicine due to their pluripotency and capacity for self-renewal. As one of the most important characteristics of a potential cell for grafting, immunogenicity has been extensively researched. Yuan *et al.* [35] reported that embryonic stem cells (ESCs) and their derivatives escaped host immune attack and survived for long periods in animal models. There are at least three reasons for this low immunogenicity. First, human ESCs express low levels of human leukocyte antigen (HLA) class I molecules and do not express HLA class II molecules in either the resting or differentiated state [36, 37]. Second, ESCs lack co-stimulatory molecules, such as CD80 and CD86 [38]. Third, ESCs suppress naive and dendritic cell-mediated T-cell proliferation in allogeneic settings [39]. Like ESCs, mesenchymal stem cells (MSCs) also have negligible immunogenicity and the capacity for immune suppression. MSCs express low levels of major histocompatibility complex (MHC) class I molecules but lack expression of MHC class II molecules and the co-stimulary molecules CD80, CD86, and CD40 [40–42]. In addition, MSCs inhibit dendritic cells [43–45], T cells [46, 47], B cells [48], natural killer cells [49, 50], and macrophages [51]. Contrary to expectation, however, some studies have found that induced pluripotent stem cells (iPSCs) derived from autologous cells are rejected by the recipient.

Zhao and colleagues [52] reported that, after reprogramming, iPSCs expressed high levels of Zg16 and Hormad, which led to immunogenicity. Moreover, the teratomas formed by these iPSCs were rejected by the recipient after syngeneic subcutaneous transplantation. In contrast, Guha *et al.* [53] found no correlation between the expression of Zg16 and Hormad and the survival of syngeneic iPSCs grafts, as neither undifferentiated syngeneic iPSCs nor differentiated cells derived from them were rejected after subrenal capsule transplantation. Similarly, Araki *et al.* [20] reported that iPSCs were no more immunogenic than ESCs. To explain this contradiction, Kaneko and Yamanaka speculated that variation in immune response may be due to the different iPSCs lines and vectors used for reprogramming [54]. The iPSCs with strong immunogenicity reported by Zhao *et al.* [52] were derived with retroviruses. Retroviral vectors can change host gene expression by integrating at transcriptional sites, resulting in the abnormal production of immunogenic proteins. Kaneko and Yamanaka thus suggested that retroviral vectors should not be used to generate iPSCs for transplantation therapy [54]. Alternatively, Fu [55] suggested that, compared with cells grafted into the subrenal capsule, cells grafted subcutaneously are exposed to more functional dendritic cells (such as Langerhans cells). Therefore, even cells with immunogenicity in other tissues (like iPSCs transformed using retroviral vectors) may not induce rejection when injected into the subrenal capsule. Thus, the subrenal capsule may not be an appropriate site for studying the immune response to minor antigens in preclinical studies. Cao *et al.* [56] speculated that iPSCs could acquire genetic and/or epigenetic defects after reprogramming, thereby generating immunogenicity. As not all descendants of iPSCs express the defects during development and differentiation, however, autologous iPSCs could show much weaker immunogenicity than allografts. If they expressed immunogenic minor antigens anomalously, however, they still could elicit immune rejection [56]. Hence, the potential immunogenicity of each iPSC-derived cell line should be tested carefully before clinical application.

## Studies of immune rejection after subretinal transplantation

In the majority of instances, donor cells survive SRS transplantation without immunosuppressive drugs. We analyzed a myriad of studies [57–70] focused on the immune reaction and/or using immunosuppressive drugs. Findings can be summarized as follows. First, none of the hosts administered immunosuppressive drugs showed immune rejection during the observation period, suggesting that immunosuppressants are effective for prevention of immune rejection of grafts within the SRS. Second, only three studies reported classic rejection at the transplant site. One found disruption of the host RPE layer at the transplant site [64], one used laser burn [60], and one used diathermy [58] during surgery, and none applied postoperative immunosuppression. Although laser application promoted migration and integration of donor cells into host retina [71], it led to focal injury of the RPE and likely breached the blood-retinal barrier, implying that the integrity of the blood-retinal barrier also plays an important role in graft preservation. Lu and colleagues [67] provided further evidence that maintenance of blood-retinal barrier integrity, rather than immunosuppression, is the critical factor for preventing rejection following SRS transplantation. In their study, they excluded eyes with a damaged blood-retinal barrier after subretinal injection and found no statistical difference in rejection rate between the immunosuppressor-treated group and the no immunosuppressor group. Indeed, both groups achieved therapeutic benefits from transplantation without immunological rejection. These observations collectively demonstrate that if the blood-retinal barrier is preserved during surgery, immunosuppressive drugs are not necessary. Note also that several of the studies used rabbits as recipients, all receiving postoperative immunosuppression. While no immune rejection was reported, the survival of the grafts was generally poor [63–65]. This may be associated with the unique retinal anatomy of rabbits. Compared with holangiotic species such as humans and rats, the rabbit retina is merangiotic [72], meaning that only part of the inner retina is supplied by retinal vessels and is more dependent, therefore, on choriocapillaries, while humans and rats possess a retinal vasculature that penetrates throughout the inner retina. Thus, the rabbit may not be the best candidate for animal investigations of retinal cell therapy.

## Surgical methods used in subretinal transplantation

A variety of surgical techniques have been developed to allow efficient SRS transplantation while protecting the blood-retinal barrier. For rodents with small eyes (rats and mice), scleral incision by a needle or blade is followed by stem cell injection through a syringe. In order to prevent reflux and improve the survival of cell grafts, several studies have used corneal puncture, which also reduces intraocular pressure. Some groups have kept the needle in the injection site for a few seconds then pulled it out slowly to maximize cell delivery to the SRS and minimize the potential for damage [73], and another created a self-sealing sclerotomy [74]. For subjects with larger eyes, such as rabbits, pigs, monkeys, and humans, an initial posterior pars plana vitrectomy has been performed to induce posterior vitreous detachment, then a syringe used to deliver the grafted cells. We suggest that the vitrectomy can reduce cell reflux and intraocular pressure. While cells are usually injected as a suspension, some groups have created new approaches to cell delivery. Kamao *et al.* [58] generated monolayer cell sheets without scaffolds using collagen gel and collagenase, while Stanzel *et al.* [63] and Hu *et al.* [75] cultured and maintained seed cells on polyester membranes and parylene substrates, respectively. In all these studies, cells were transplanted as a layered structure rather than as a suspension. Kundu *et al.* [76] demonstrated that suspended cells injected in the SRS could integrate into the host retina, but survival was poor due to loss of cells by reflux, while cell layer transplantation allowed for greater cell retention. If cell layers are transplanted, any scaffold used must be biocompatible and non-immunogenic but strong and flexible enough to withstand surgical manipulation [76].

However, all current clinical trials registered by the US National Institutes of Health use suspended cells for subretinal transplantation. Most use MA09-hRPE cells, which are fully differentiated from human ESCs, while one is using OpRegen cells, which are RPE cells derived from human ESCs (NCT02286089). Two groups are using human central nervous system stem cells (NCT01632527, NCT02137915), another team human retinal progenitor cells (NCT02464436), and another autologous bone marrow-derived stem cells (NCT01920867). In Japan (RIKEN), iPSCs are currently being used in clinical trials to treat age-related macular degeneration.

## Microglial activation in the retina following stem cell transplant

While most studies have found no evidence of graft rejection, such as leakage of fluorescein on fluorescein angiography or immune cell infiltration at transplantation sites, long-term graft survival rates are often poor. This poor survival is a major hindrance to clinical application and has thus received extensive experimental attention. Singhal *et al.* [77] observed substantial microglial accumulation at the site of cell injection, which inhibited Müller stem cell migration, and Bull *et al.* [78] reported that, in spite of an immunosuppressive regimen, Müller stem cells transplanted into the vitreous or SRS were eventually rejected due to attack by microglia/macrophages.

Microglia are the resident cells mediating innate immunity in the retina. There are two distinct microglial populations: the perivascular macrophages situated within the glial limitans of the inner retinal vasculature and the ramified retinal microglia within the tissue parenchyma [79, 80]. In the developing human retina, microglia are present in the developing nerve fiber as well as the ganglion cell, inner plexiform, and outer plexiform layers [81]. Ramified microglial cells are characterized by small, slender cell bodies, with long, radial, and highly dynamic protrusions [82]. This special morphology allows the entire microglia population to cover every part of the retina and contribute to tissue homeostasis without activation [83]. However, the situation changes when microglia are activated by nerve degeneration [82], inflammation [84, 85], traumatic nerve lesion [86], or excessive light overexposure [87]. Activated microglia show directed polarity and rapidly migrate toward the region of damage. In the activated state, they have dual capacity to modulate neurogenesis, both by enhancing progenitor cell proliferation and by inhibiting neurosphere generation and the extent of differentiation [88]. Activated microglia also exert complex effects on immunity and cell survival by secreting various cytokines, such as interleukin (IL)-1β, IL-10, tumor necrosis factor (TNF)-α, and IL-6 [89]. While IL-10 is generally considered anti-inflammatory, immunosuppressive, and neuroprotective [90], IL-1β, IL-6, and TNF-α are pro-inflammatory and neurotoxic [84].

## Treatments for moderating microglial activation

Endogenous glucocorticoids, such as triamcinolone, prednisolone, and dexamethasone, are well known suppressors of the innate immune response [91]. Glucocorticoids can suppress cytokine-mediated microglia proliferation [92] and accumulation [93], and downregulate cytotoxic molecules such as nitric oxide [94], TNF, IL-6 [95], and glutamate [96]. Singhal *et al.* [93] reported that the survival of transplanted cells was significantly enhanced by triamcinolone. The novel resveratrol analogue RV09 (5-[2-(4-bromothiophen-2-yl)vinyl]benzene-1,3-diol) can also control microglial activation and cytotoxicity. Meng *et al.* [97] found that RV09 inhibited lipopolysaccharide-induced nitric oxide and TNF-α production in microglia. In addition to glucocorticoids and RV09, minocycline suppressed microglial activation [98–101] and prevented neuronal loss by inhibiting inducible nitric oxide synthase induction [102], caspase expression [103], and cytochrome c release [104]. However, while 10 μg/ml or lower did not affect neural progenitor cell (NPC) survival and proliferation, higher minocycline concentrations (20 and 40 μg/ml) impaired NPC differentiation in culture [105].

## Effective ways to improve graft survival following subretinal transplantation

The studies cited above suggest several ways to mitigate the immune response following transplantation. The most important is to improve surgical skills and procedures to maintain blood-retinal barrier integrity. Drugs such as glucocorticoids, RV09, and minocycline that can inhibit proliferation and activation of microglia are also beneficial. If the blood-retinal barrier is ruptured during surgery, immunosuppressive drugs such as cyclosporine A may be necessary. However, these agents should be used with caution. Skardelly *et al.* [106] found that even at the minimum effective concentrations, all immunosuppressants tested (cyclosporine A, everolimus, mycophenolic acid, and prednisolone) reduced the proliferative capacity of human NPCs, especially cyclosporine A and mycophenolic acid, and altered their NAD(P)H-dependent metabolic activity. Moreover, mycophenolic acid treatment induced apoptotic death. Alternatively, cell death rate, neurogenesis, gliogenesis, and cell migration were unaffected by these agents. Rota *et al.* [107] suggested that transient immunosuppression was sufficient for long-term survival of human NPCs and engraftment. Wenkel *et al.* [27] also found that the blood-retinal barrier was fully reformed 21 days after intravenous injection of sodium iodate. Hence, to reduce the side effects of traditional immunosuppressants, drugs can be withdrawn after a month. Additionally, injection of grafts combined with chondroitinase ABC caused a dramatic increase in the migration of Müller stem cells into all retinal cell layers [77] and improved synapse formation of transplanted photoreceptor precursors with host neurons [108]. This may be explained by the repression of synaptogenesis [109], stem cell migration, and integration into the damaged retina by microglial deposition of chondroitin sulfate proteoglycans. More specific immunosuppressive strategies have also been developed to improve graft survival. Pearl *et al.* [110] show that blocking leukocyte co-stimulatory molecules, such as cytotoxic T-lymphocyte-associated antigen 4 (CTLA4-Ig), anti-CD40 ligand, and anti-lymphocyte function-associated antigen 1, permitted long-term engraftment of allogeneic ESCs, mitigated xenogeneic immune rejection of both undifferentiated and *in vivo* differentiated ESCs, and prevented rejection following allogeneic and xenogeneic transplantation of iPSCs. Rong *et al.* [111] established knock-in human ESCs constitutively expressing CTLA4-Ig and PD-L1 before and after differentiation, and showed that human ESC-derived allografts could be implanted without the need for systemic immune suppression.

## Conclusion

Although both the SRS and stem cells have low immunogenicity, therapeutic stem cells in various states of differentiation and delivered via different transplantation protocols show distinctive survival, differentiation, and migration capacities in the host. First, cell survival after transplantation depends on technical aspects of graft preparation, including the harvesting technique, cell purity, and tissue storage methods. Second, the state of the host also influences transplant success, as better migration and integration of stem cells have been observed when neural progenitors are transplanted into immature or injured retina [112–114]. The anatomy of the retina also influences the survival of the graft, so host species is important for experimental studies and clinical translation. Appropriate surgical methods are also paramount. Cells injected into the SRS migrate in the retina better than cells injected into the vitreous cavity. Anterior chamber paracentesis or vitrectomy can prevent graft leakage and mitigate excessive intraocular pressure during surgery. Chondroitinase ABC can be used to increase the migration of stem cells into all retinal cell layers. Postoperational treatment also influences graft survival. As microglia are activated after transplantation, anti-microglial drugs could be of great benefit. When the blood-retinal barrier is broken, immunosuppressants are needed, at least until the barrier has reformed. These drugs should be withdrawn as early as possible, however, to mitigate suppressive effects of these agents on cell survival, proliferation, and/or migration.

## Abbreviations

CTLA4-Ig: Cytotoxic T-lymphocyte-associated antigen 4
ESC: Embryonic stem cell
HLA: Human leukocyte antigen
IL-1: Interleukin
iPSC: Induced pluripotent stem cell
MHC: Major histocompatibility complex
MSC: Mesenchymal stem cell
NPC: Neural progenitor cell
PD-L1: Program death-1
RPE: Retinal pigment epithelium
RV09: 5-[2-(4-bromothiophen-2-yl)vinyl]benzene-1,3-diol
SRS: Subretinal space
TNF: Tumor necrosis factor
